# Intracranial pressure is a physiological stress that determines sympathetic nervous system activity

**DOI:** 10.1186/2045-8118-12-S1-O56

**Published:** 2015-09-18

**Authors:** Eric Albert Schmidt, Fabien Despas, Anne Pavy Le Traon, Marek Czosnyka, John Douglas Pickard, Kamal Rahmouni, Atuk Pathak, Jean Michel Senard

**Affiliations:** 1 Institut National de la Sante et de la Recherche Médicale, UMR-1048, Institut des Maladies Metaboliques et Cardiovasculaires, Toulouse, France; 2 Department of Neurosurgery and Institute for Neurosciences, CHU and Toulouse University, Toulouse, France; 3 Department of Clinical Pharmacology, CHU and Toulouse University, Toulouse, France; 4 Department of Neurology and Institute for Neurosciences, CHU and Toulouse University, Toulouse, France; 5 Academic Neurosurgery, Department of Clinical Neurosciences, University of Cambridge, Cambridge, UK; 6 Departments of Pharmacology and Internal Medicine, University of Iowa Carver College of Medicine, Iowa City, Iowa, USA

## Introduction

The interplay between CSF dynamics, intracranial pressure (ICP), arterial blood pressure (ABP) and sympathetic nervous system (SNS) remains poorly understood. We know that raised ICP reduces CPP (i.e. ABP-ICP) and blood delivery to the brain. Massive ICP rise is also known to produce an increase in ABP termed the Cushing response, a terminal event occurring in extreme pathological conditions of brainstem ischaemia leading to a sympatho-adrenal response. Hence the Cushing response is not activated in a normal patient and is not involved in the pathophysiology of NPH. However, it is still debated whether the Cushing response is an acute pathological response to brain ischemia or part of an important physiological reflex mechanism. Indeed clinical and experimental studies suggest that modest ICP increase modulates systemic hemodynamics probably via the SNS. We hypothesize that modest ICP changes drive sympathetic activity. Using state-of-the-art technique to measure SNS, we performed two different sets of experiments in humans and mice. In both species during controlled hydrostatic modest ICP increase and decrease, SNS was measured directly by microneurography and indirectly by heart rate variability analysis (HRVA).

## Methods

In 10 patients suspected of NPH, ICP was measured and increased during lumbar infusion study. HR and ABP were non-invasively monitored. Muscle sympathetic nerve activity (MSNA) was recorded. In 15 anesthetized mice, ICP was measured and intraventricular infusion was performed. HR and ABP were invasively monitored. Renal sympathetic nerve activity (RSNA) was recorded. In human and mice, HRVA allows calculation of indices gauging sympathetic and parasympathetic indices.

## Results

In humans, modest increase in ICP was associated with a parallel increase in MSNA (e.g. 7 mmHg ICP rise increases SNS-activity by 17%). ABP was stable. Similarly in mice, modest rise in ICP increased RSNA. In both species ICP drop significantly reduced MSNA and RSNA. HRVA confirmed that modest rise in ICP augments sympathetic indices in humans and mice.

## Conclusions

Using gold-standard measurement of SNS, we demonstrate in both species that ICP drives efferent SNS outflow. ICP is not only a determinant of CPP but also a physiological stressor that influences and reversibly modulates SNS activity, even at relatively low values. We demonstrate a new physiological link between ICP and SNS activity which may represent an important highly regulated circuit. Our findings strongly suggest the presence of a novel intracranial baroreflex. It represents a paradigm shift in physiology of the heart-brain cross-talk and in the pathophysiology of hydrocephalus. CSF related diseases might participate to sympathetically-driven medical disorders.

